# Obstetrics

**Published:** 1879-04

**Authors:** 


### OBSTETRICS.

CASE OF DESQUAMATION OF THE EPIDERMIS IN A CHILD BORN ALIVE.—M. Charrier. (*L'Union Médicale*, Dec. 28, 1878.)

The following very rare case was reported by M. Charrier to the Société de Médecine. On July 9, 1878, I was called to a woman pregnant with her second child. The pregnancy had been normal, with the exception of a bronchitis treated by Dr. Millard. At one o'clock the pains which had occurred at rare intervals since morning, became more frequent, and I was called, arriving at half-past three. I found the lady already suffering the pains of expulsion, although the uterine orifice appeared to be only the size of a franc piece. The pains continued more and more violent, and at five and a half, a final pain of extreme force and duration expelled the head from the uterine orifice and beyond the vulva. The child was born with two circles of the cord around the neck, one of the loops passing under the right axilla and over the left shoulder like a scarf. The length of the cord was 83 centimeters. Half asphyxiated, pale as wax, it was only after stimulation for a minute or two with friction to the child that the child uttered its first cry. But what especially attracted my attention was the greenish red color of the umbilical cord, which was flattened like a ribbon, and appeared to belong to a dead-born child, long macerated in the waters of the amnion. Moreover, the entire epidermis of the body came away on the slightest friction, as in a macerated fœtus dead six or eight days. The epidermis of the foot was detached exactly like a glove.

The next day the epidermis had all fallen, except in two or three small places on the left leg, the back and the right arm. The child then had the natural color and temperature; it sucked with vigor, and, since, its health has been good.

I confess I have never seen a similar case; I have seen many cords greenish and withered, but never, in a living child, a desquamation like that which occurs in a fœtus long dead and macerated in the amniotic fluid.

I believe that if the labor had been delayed two or three days, the child would have been born dead; but there a question in legal medicine presents itself: Can the fœtus, then, *before death*, become macerated in the waters of the amnion? Upon what does this desquamation of the entire epidermis depend? No eruptive disease, measles or scarlatina had been observed about the mother during her pregnancy. I remarked at the beginning that her pregnancy had been normal, except a bronchitis. To what, then, can we attribute the pathological state of the child? I will add, the membranes were very friable, and the same color as the cord, that is, reddish and greenish. The amniotic fluid had no odor. The placenta was healthy. It is evident that the fœtus suffered *in utero*, but the semi-asphyxiated state in which it was born is explained by the winding of the cord around the neck and chest. But this hindrance to circulation could not be the cause of the *macerative desquamation* before birth.

